# Bioinformatics Analysis of Competing Endogenous RNA Network and Immune Infiltration in Atrial Fibrillation

**DOI:** 10.1155/2022/1415140

**Published:** 2022-07-16

**Authors:** Xing Liu, Ke Peng, Guoqiang Zhong, Mingxing Wu, Lei Wang

**Affiliations:** ^1^Department of Cardiology, Xiangtan Central Hospital, Xiangtan, Hunan 411100, China; ^2^Department of Spine Surgery, The Second Xiangya Hospital, Central South University, Changsha, Hunan 410011, China; ^3^Department of Cardiology, Guangxi Cardiovascular Institute, The First Affiliated Hospital of Guangxi Medical University, Guangxi 530000, China

## Abstract

**Background:**

There is still no clear understanding of the pathogenesis of atrial fibrillation (AF). For this purpose, we used integrated analysis to uncover immune infiltration characteristics and investigated their relationship with competing endogenous RNA (ceRNA) network in AF.

**Methods:**

Three AF mRNA data sets (GSE14975, GSE79768, and GSE41177) were integrated using the SVA method from Gene Expression Omnibus (GEO). Together with AF circRNA data set (GSE129409) and miRNA data set (GSE70887) from GEO database, we built a ceRNA network. Then hub genes were screened by the Cytoscape plug-in cytoHubba from a protein-protein interaction (PPI) network. As well, CIBERSORT was employed to investigate immune infiltration, followed by Pearson correlation coefficients to unravel the correlation between AF-related infiltrating immune cells and hub genes. Ulteriorly, circRNA-miRNA-mRNA regulatory axises that could be immunologically related to AF were obtained.

**Results:**

Ten hub genes were identified from the constructing PPI network. The immune infiltration analysis revealed that the number of monocytes and neutrophils was higher, as well as the number of dendritic cells activated and T cells regulatory (Tregs) was lower in AF. Seven hub genes (C5AR1, CXCR4, HCK, LAPTM5, MPEG1, TLR8, and TNFSF13B) were associated with those 4 immune cells (*P* < 0.05). We found that the circ_0005299–miR-1246–C5AR1 and circRNA_0079284-miR-623-HCK/CXCR4 regulatory axises may be associated with the immune mechanism of AF.

**Conclusion:**

The findings of our study provide insights into immuno-related ceRNA networks as potential molecular regulators of AF progression.

## 1. Introduction

Atrial fibrillation (AF) is a common arrhythmia, increasing with age, reaching 7.5% or more in people older than 80 [1]. In addition, the risk of embolic stroke, heart failure, and mortality increase with AF [1, 2]. Although treatment strategies of AF have advanced dramatically in recent years, their efficacy is not ideal, especially in the radiofrequency ablation treatment of persistent AF (PAF) [3]. The reason is the incomplete knowledge of the AF mechanisms. To develop more effective treatments for AF, we therefore need to gain a deeper understanding of the molecular and cellular mechanisms involved in AF.

CircRNAs are new RNA molecules with unique biological functions that can act as sponges for microRNAs (miRNAs) to bind competitively and regulate parental genes expression [4, 5]. A growing body of evidence suggests that circRNA-miRNA-mRNA regulatory axis are involved in cardiovascular disease's pathogenesis. CiRS-7, for example, is useful as a sponge for miRNA-7a, which promotes myocardial apoptosis via inhibiting PARP and SP1 expression [6]. The competitive binding of heart-related circRNA (HRCR) with endogenous miR-223 increases the expression of ARC gene, thereby inhibiting heart failure and cardiac hypertrophy [7]. Further, circRNA_000203 promote fibrosis-associated gene expression by inhibiting miR-26b-5p targets, contributing to myocardial fibrosis [8]. Hence, competing endogenous RNA (ceRNA) networks may shed light on AF pathophysiology. Studies have also shown that inflammation and the immune response it triggers are crucial to the development of AF [9, 10]. However, rarely have studies examined the relationship between immune cells infiltration and ceRNA networks in atrial tissue of AF patients.

**Figure 1:**
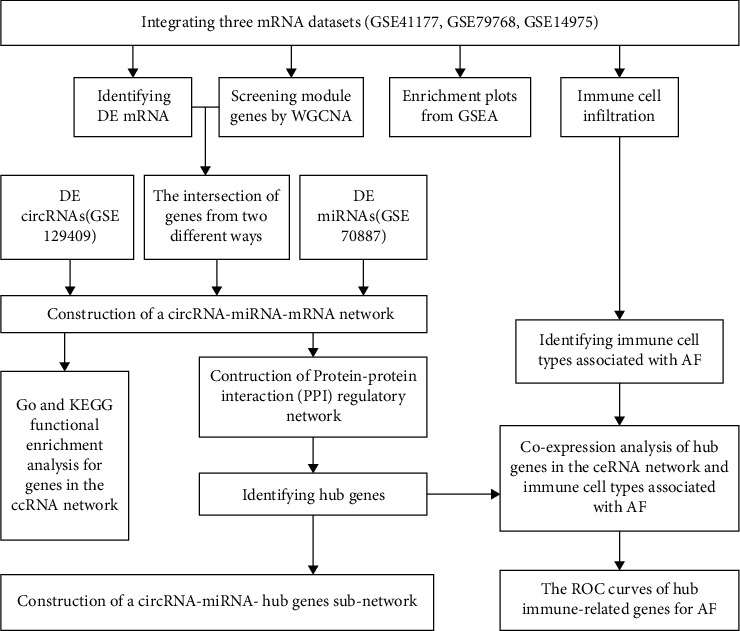
Flow diagram of bioinformatics analysis.

In our study, on the one hand, we integrated three PAF data sets by the SVA method and used weighted gene co-expression network analysis (WGCNA) and differential expression analysis to identify common genes (CGs). Differentially expressed (DE) miRNAs and DE circRNAs in PAF were identified from GSE70887 and GSE129409 data set, respectively. Together with CGs, ceRNA network was built based on circRNA-miRNA pairs and miRNA-mRNA pairs. Hub genes were then filtered using the Cytoscape plug-in cytoHubba by analyzing protein-protein interaction (PPI) networks. On another hand, CIBERSORT was used to study immune infiltration in AF [11]. In the following analysis, Pearson correlation coefficients were used to determine the correlation between AF-related infiltrating immune cells and hub genes. Finally, we gained novel insight into the mechanisms that govern the progression of AF by the analysis of immune-related ceRNA networks. The study flowchart is shown in Figure 1.

## 2. Materials and Methods

### 2.1. Data Acquisition

Three AF mRNA data sets (GSE14975, GSE79768, and GSE41177), AF circRNA data set (GSE129404), and AF miRNA data set (GSE70887) were downloaded from gene expression omnibus (GEO) [12] database. Three left atrial appendage samples from PAF patients and three sinus rhythm (SR) controls were included in GSE129409, while four atrial appendage samples from PAF patients and two SR controls were contained in GSE70887. Among the GSE14975, GSE79768, and GSE41177, left atrial appendage tissue was obtained from five PAF patients and five SR controls, seven PAF patients and six SR controls, and sixteen PAF patients and three SR controls, respectively.

All data sets originated from a free open-access database on the Internet; thus, this study does not require ethical approval and patient consent.

### 2.2. Data Processing and Gene Set Enrichment Analysis (GSEA)

In order to transform gene probe IDs to gene symbol codes, the series matrix files were processed by ActivePerl 5.24.2 software (https://www.activestate.com/products/perl/). In the three mRNA data sets, the data in GSE14975 data set were transformed into log base 2 data by affy package in R [13]. The sva package's combat function was applied to remove batch effects and other undesired variation between the three mRNA microarray data sets after merging all data [14]. In the end, R software's “limma” package contains the “normalizeBetweenArrays” function [15], which normalized expression values. Gene set enrichment analysis (GSEA) is a computational algorithm for determining whether a predefined set of genes exhibit consistently significant differences between two states [16]. In a GSEA, the sequenced genes of AF and SR samples are analyzed after imputting gene annotation files, reference function sets, and all the gene data from both samples. The pathways enriched in each phenotype were analyzed based on nominal *p* value and normalized enrichment score (NES).

### 2.3. Weighted Gene Co-Expression Network Analysis (WGCNA) Construction and Identification of Modules

Gene co-expression network was constructed using the integrated data set with the help of a system biology approach of WGCNA [17]. The soft thresholding power *β* was set as 5 and 20 and was selected using the function pickSoftThreshold. In order to classify genes with similar expression profiles into gene modules, average linkage hierarchies were clustered according to topological overlap matrix (TOM)-based difference measure, and the minimum size (gene group) of the genes dendrogram was 50 [18]. Finally, module membership (MM), gene significance (GS), and module-trait correlations analyses were conducted. *P* < 0.05 was defined as statistically significant module.

### 2.4. Identification of Differential Expression of circRNAs, miRNAs, and mRNAs

In this study, DE mRNAs, DE miRNAs, and DE circRNAs were screened using the Limma package in R. The integrated data set was analyzed with |log_2_ Fold change | > 0.5 and *p* value < 0.05 set as the cut-off point for selecting DE mRNA. For analysis of GSE70887, |log_2_ Fold change | > 1 and *p* value < 0.05 were used as criterion for selecting DE miRNA. For analysis of GSE129409, |log_2_ Fold change | > 3 and *p* value < 0.05 were used as criterion for selecting DE circRNA. The “ggplot2” and “pheatmap” packages of R software were used to create volcano maps and heatmaps for DE mRNA, DE miRNAs, and DE circRNAs. Common genes (CGs) are the intersection of DE mRNA identified from the integrated mRNA data set and the genes found in yellow module.

### 2.5. Construction of a circRNA-miRNA-mRNA Regulatory Network

CircRNAs information can be found in CircBase (https://www.circb ase.org/) [19]. The cancer-specific circRNA database (CSCD, https://gb.whu.edu.cn/CSCD/) [20] was able to predict target miRNAs for each DE circRNA. Then, we gathered miRNAs that overlapped both DE and predicted miRNAs and used TargetScan database [21] to predict targeted genes. Next, those targeted genes were considered as candidate targets and overlapped with CGs. Lastly, we constructed a ceRNA regulatory network of AF and visualized it using Cytoscape version 3.8.0.

### 2.6. GO and KEGG Functional Enrichment Analysis

To assess the functional annotations of genes in ceRNA regulatory network, GO and KEGG functional enrichment analysis was carried out based on the “clusterprofiler,” “ggplot2 Goplot,” “digest,” “org.Hs.eg.db,” and “enrichplot” packages of R/Bioconductor. Statistically significant values were defined as *p* value < 0.05.

### 2.7. Construction of PPI Regulatory Network and Identification of Hub Genes

Using the STRING datebase (https://string-db.org) [22], a PPI network was constructed for these genes in the ceRNA network, and a minimum interaction score of 0.4 was considered the cutoff point. CytoCope 3.8.0 software was utilized to visualize the PPI network, and the Maximal Clique Centrality (MCC) arithmetic of the Cytoscape plug-in cytoHubba was used to filter hub genes in the PPI network. Finally, a circRNA-miRNA-hub gene subnetwork was constructed. Boxplot maps representing differential expression of circRNAs, miRNAs, and mRNAs in their microarray data sets in the circRNA-miRNA-hub gene subnetwork were generated with the help of “reshape2” and “ggpubr” packages of R software.

### 2.8. Immune Cell Infiltration Analysis

The integrated data set was analyzed using CIBERSORT in R software to compute the relative proportion of infiltrating immune cells in AF, and the samples were filtered using *P* < 0.05. A principal component analysis (PCA) was performed on immune cell infiltration using the “ggplot2” package. The 22 types of infiltrating immune cells were subjected to a Spearman correlation analysis using R software, and we generated a correlation heatmap with the help of “Corrplot” package in R for visualizing the results. The expression of 22 immune cells was compared and visualized using “vioplot” package between PAF and SR samples.

### 2.9. Correlation Analysis between Hub Genes and Infiltrating Immune Cells Associated with AF

A Pearson correlation coefficient was applied to examine the relationship between hub genes and AF-related infiltrating immune cells, which was visualized with the “ggpubr” package of R.

### 2.10. Diagnostic Analysis of Hub Immune-Related Genes for AF

For the purpose of determining the effectiveness of hub immune-related genes in predicting AF, receiver operator characteristic (ROC) curve analysis was conducted with the help of “pROC” package.

## 3. Results

### 3.1. Enrichment Analysis of Merged Expression Data through GSEA

**Figure 2:**
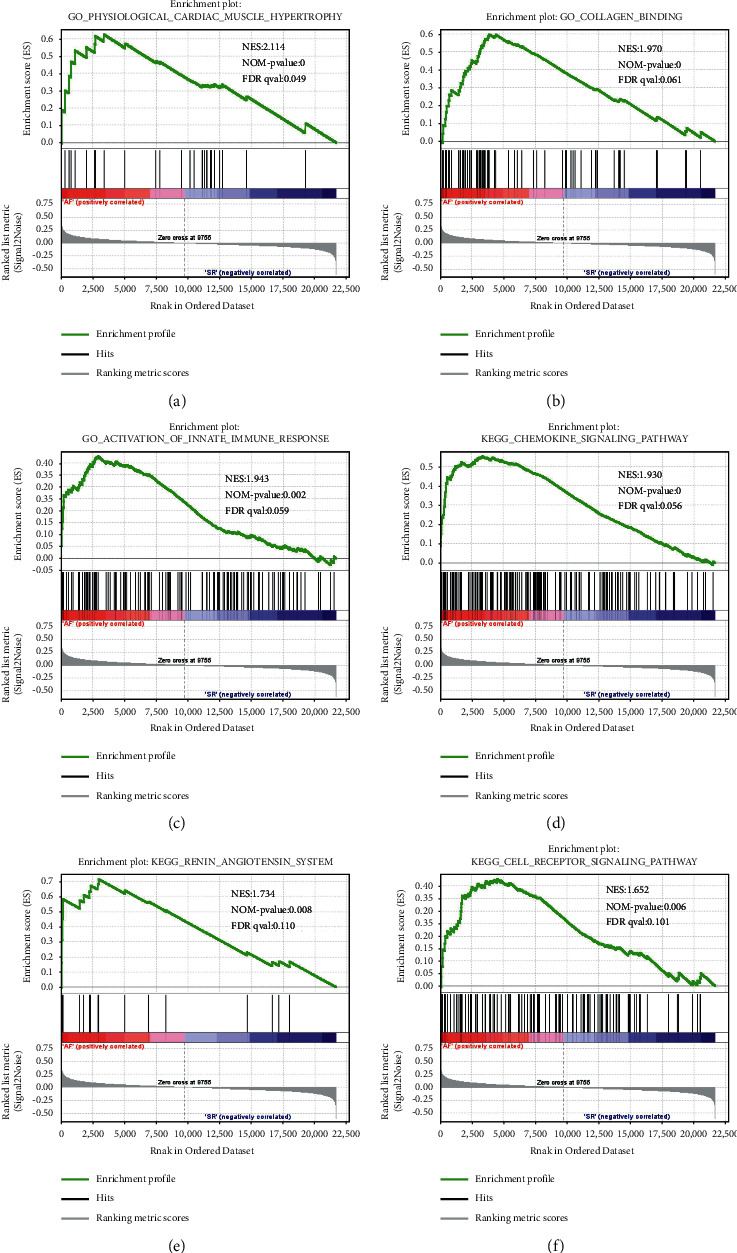
Enrichment analysis of mRNA integrated data set through gene set enrichment analysis (GSEA). GSEA results showing physiological cardiac muscle hypertrophy (a), collagen binding (b), activation of innate immune response (c), chemokine signaling pathway (d), renin angiotensin system (e), and T cell receptor signaling pathway (f) are differentially enriched in atrial fibrillation.

GSEA was applied to analyze the significant difference between AF and SR groups for the integrated data set. The enrichments for upregulated gene sets in the significant order (size of NES) were related to physiological cardiac muscle hypertrophy (GO) (Figure 2(a)), collagen binding (GO) (Figure 2(b)), activation of innate immune response (GO) (Figure 2(c)), chemokine signaling pathway (KEGG) (Figure 2(d)), renin angiotensin system (KEGG) (Figure 2(e)), and T cell receptor signaling pathway (KEGG) (Figure 2(f)).

### 3.2. Identification of Gene Co-Expression Networks and Modules

**Figure 3:**
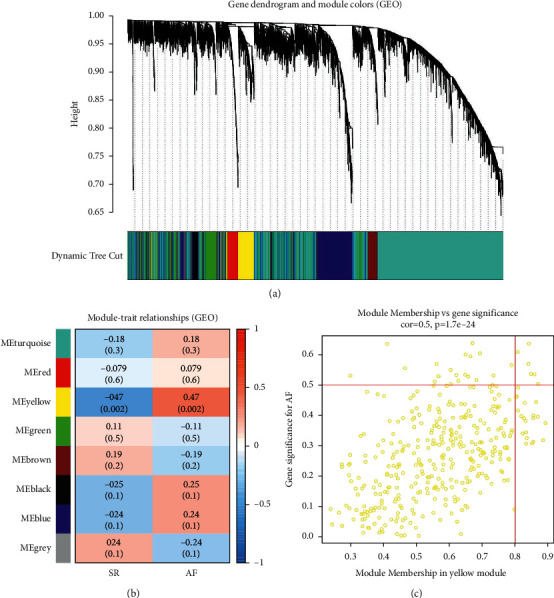
Construction of the weighted co-expression network and module analysis. (a) Differentially expressed genes represented by different colors under the gene tree. (b) Module-trait relationships. The yellow module correlated significantly with atrial fibrillation. (c) The scatter plots show the correlations between the yellow modular gene and atrial fibrillation.

Using the WGCNA package, gene co-expression network was built from the integrated gene data set to identify functional clusters in AF patients. Eight modules were excavated after setting the power to 5 (Figures 3(a)). In Figure 3(b), the module-trait relationships are illustrated, showing that the yellow module has the greatest relationship with AF (*r* = 0.47, *p*=0.002), encompassing 365 genes. The greatly significant correlation between GS and MM indicates that genes in the yellow module are greatly associated with AF (cor = 0.5, *p*=1.7*e* − 24) (Figure 3(c)).

### 3.3. Identification of DE circRNAs, DE miRNAs, DE mRNAs, and CGs

**Figure 4:**
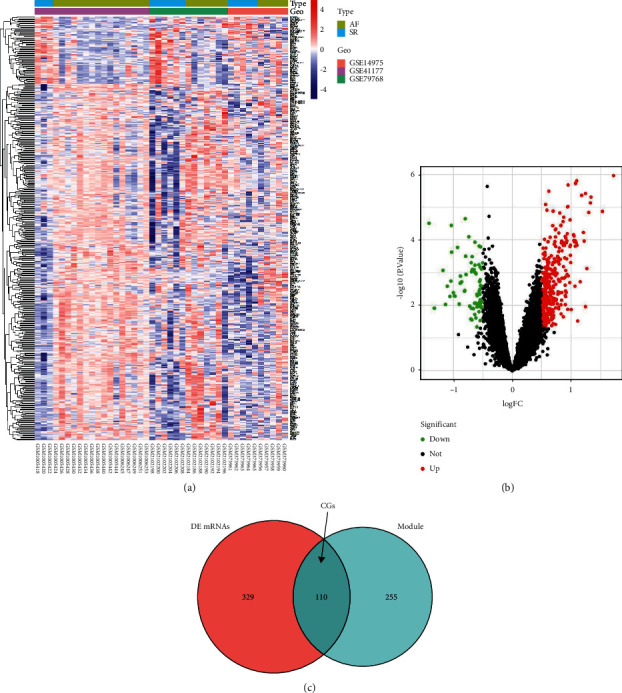
Identification of DE mRNAs in atrial fibrillation from the integrated mRNA data set. Heat map (a) and Volcano plot (b) for the DE mRNAs. (c) A total of 110 overlapping genes defined as common genes (CGs) between the DE mRNAs and the genes found in the most significant module were identified.

**Figure 5:**
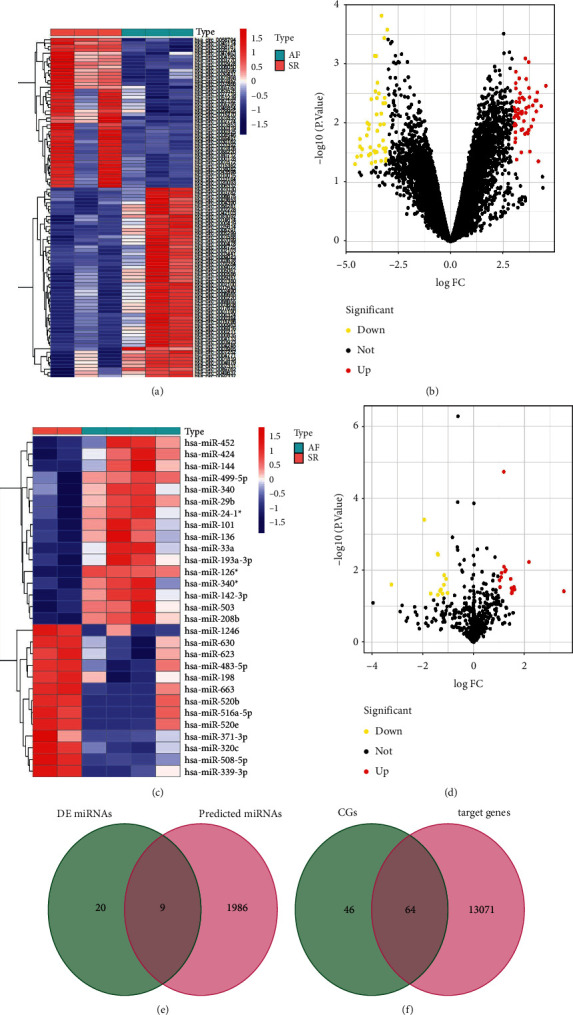
Identification of DE circRNAs, DE miRNAs in atrial fibrillation. Volcano plots (a) and heat map (b) of DE circRNAs between atrial fibrillation and sinus rhythm group. Volcano plots (c) and heat map (d) of DE miRNAs between atrial fibrillation and sinus rhythm group. (e) A total of 9 overlapping miRNAs between the DE miRNAs and the predicted miRNAs were identified. (f) A total of 64 overlapping mRNAs between the common genes (CGs) and the targeted genes were screened.

In the merged mRNA data set, a total of 439 DE mRNAs were screened in AF (Figures 4(a), 4(b)). Then, CGs were defined as the intersection of DE mRNAs from the integrated mRNA data set and genes from yellow module (Figure 4(c)), including 110 genes. Totally, 103 DE circRNAs were screened in the circRNA expression profile data (Figures 5(a), 5(b)). In addition, 29 DE miRNAs were identified in the miRNA expression profile data (Figures.  5(c), 5(d)).

### 3.4. Construction of ceRNAs Regulatory Networks in AF

**Figure 6:**
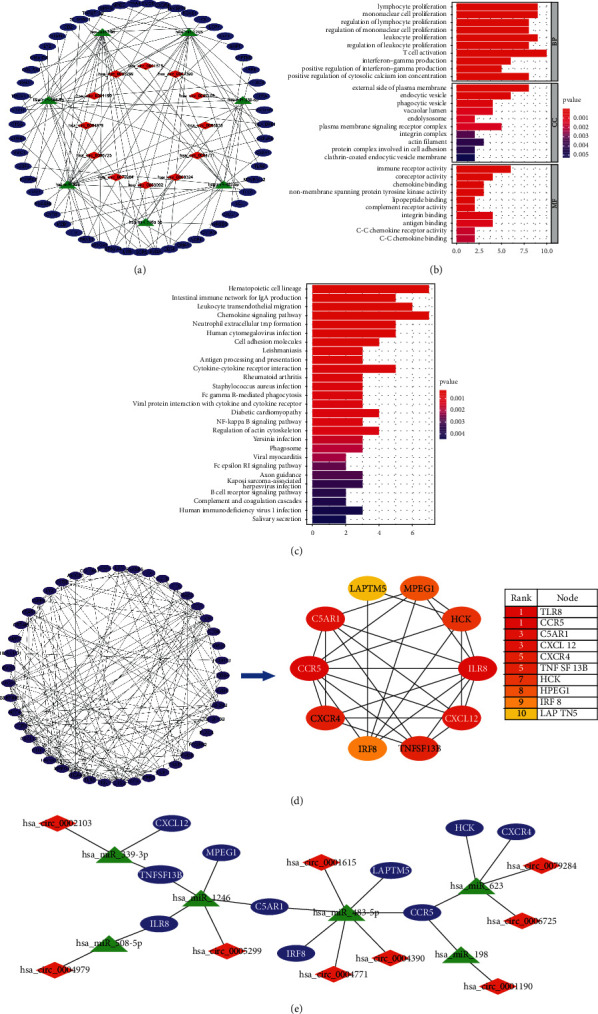
Constructing and analyzing the ceRNA network. (a) CeRNA network of circRNA-miRNA-mRNA interactions in atrial fibrillation. (b) An analysis of the GO enrichment of mRNAs in the ceRNA network. (c) An analysis of the KEGG pathway enrichment of mRNAs in the ceRNA network. (d) PPI network construction and hub gene selection. (e) CircRNA-miRNA-hub gene subnetwork construction. Upregulated circRNAs are indicated by red diamonds, downregulated miRNAs by green triangles, and upregulated mRNAs by blue ovals.

Fifteen DE circRNAs were not found in the CSCD database. Based on this database, 1,995 targeted miRNAs were predicted from the remaining 88 DE circRNAs. In the next step, 9 miRNAs were obtained through the intersection of DE miRNAs and predicted miRNAs (Figure 5(e)). Using the TargetScan database, 9 miRNAs predicted 13,135 potential targets. Then, 64 mRNAs were acquired by the intersection of CGs and predicted target genes (Figure 5(f)). Finally, the ceRNA network associated with AF was constructed (Figure 6(a)).

### 3.5. Functional Enrichment Analyses for mRNAs in the ceRNAs Network

Go functional enrichment analysis revealed that those genes in the circRNA-miRNA-mRNA ceRNA network were primarily involved in biological process (BP) terms, including “T cell activation,” “lymphocyte proliferation.” In the cell component (CC) ontology, those genes were mainly enriched in “external side of plasma membrance,” “endocytic vesicle.” Molecular function (MF) analysis indicated that those genes were significantly enriched in “immune receptor activity,” “coreceptor activity” (Figure 6(b)). The KEGG pathway of those genes were primarily involved in “hematopoietic cell lineage,” “chemokine signaling pathway,” “leukocyte transendothelial migration” (Figure 6(c)).

### 3.6. PPI Network Analysis

**Figure 7:**
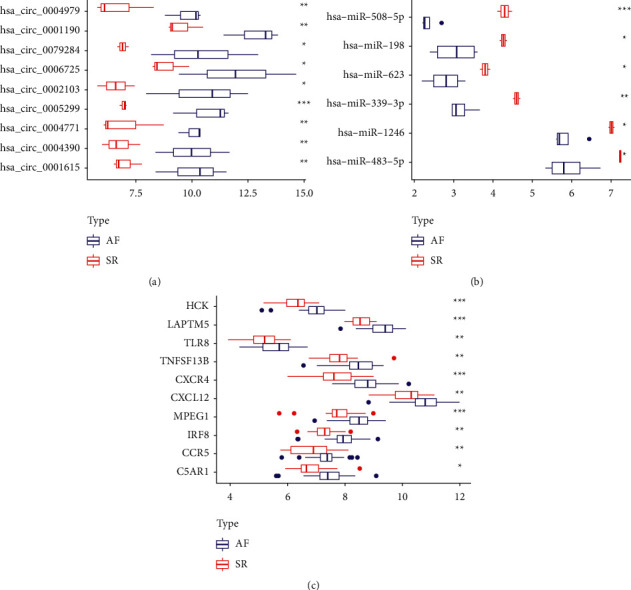
Differential expression of circRNAs (a), miRNAs (b), and mRNAs (c) in their microarray data sets in the circRNA-miRNA-hub gene subnetwork. ^*∗*^*P* < 0.05, ^*∗∗*^*P* < 0.01, and ^*∗∗∗*^*P* < 0.001.

Using STRING database, 55 mRNAs in ceRNAs network were constructed into a PPI network consisting of 45 nodes and 160 edges after removing unconnected nodes (Figure 6(d)). To explore and construct the crucial circRNA-miRNA-hub genes regulatory axis in the progression of AF, the MCC algorithm was used to identify hub genes in the PPI network. Through the MCC scoring method, the top ten genes were defined as hub genes (Table 1), which were C–C chemokine receptor type 5 (CCR5), C-X-C chemokine receptor type 4 (CXCR4), Toll-like receptor 8 (TLR8), stromal cell-derived factor 1 (CXCL12), C5a anaphylatoxin chemotactic receptor 1 (C5AR1), hematopoietic cell kinase (HCK), tumor necrosis factor ligand superfamily member 13B (TNFSF13B), interferon regulatory factor 8 (IRF8), macrophage gene 1 protein (MPEG1), and lysosomal-associated transmembrane protein 5 (LAPTM5), respectively (Figure 6(d)). And those hub genes were upregulated in AF. Subsequently, a circRNA-miRNA-hub gene subnetwork was showed in Figure 6(e).  Figure 7 shows the differential expression of each molecule in the ceRNA subnetwork in the microarry data sets. The basic information of the 9 circRNAs in the ceRNA subnetwork are listed in Table 2.

### 3.7. Immune Infiltration Analyses

**Figure 8:**
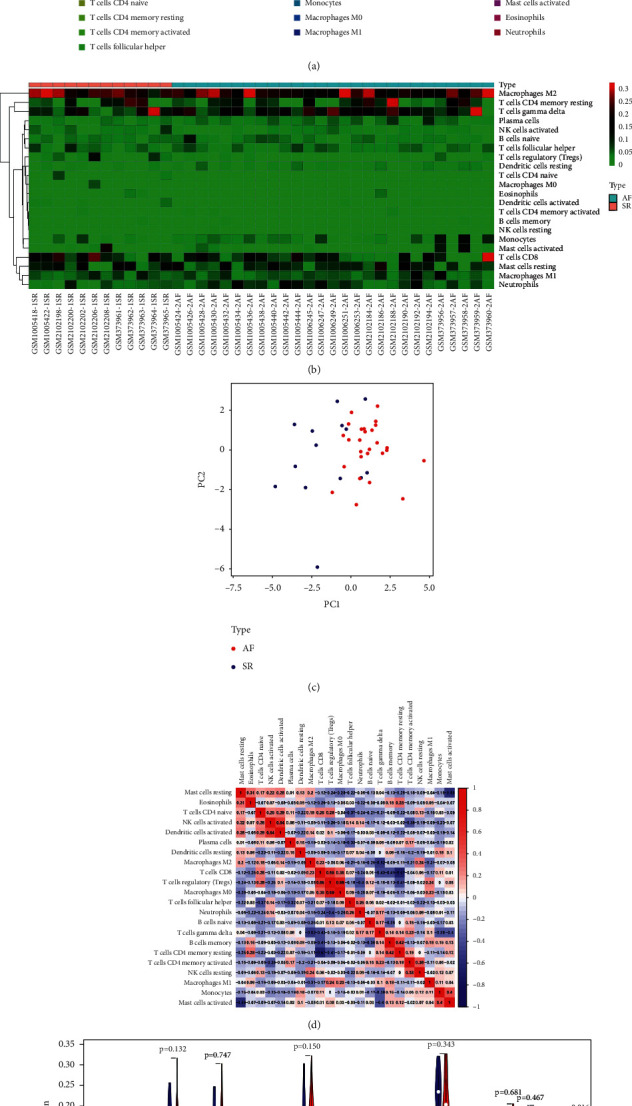
Analyses of immune infiltration in atrial fibrillation. The relative percentage (a) and heatmap (b) of 22 types of immune cells. (c) Principal component analysis of immune cells in atrial fibrillation and normal controls. (d) The correlation of the infiltration of innate immune cells. (e) Comparison of 22 immune cell subtypes between patients in atrial fibrillation and controls.

Using the CIBERSORT algorithm, bar plots and heat maps display the relative proportion of the 22 types of immune cells detected in each sample (Figures 8(a), 8(b)). Using PCA, the immune cells in the atrial tissues of AF patients and SR revealed distinct group bias clustering and individual differences (Figure 8(c)). Correlation analysis between infiltrating immune cells and T cells regulatory (Tregs) was positively related to macrophages M0 (*r* = 0.69) and negatively correlated to neutrophils (*r* = 0.40) and the activated dendritic cells were positively related to activated NK cells activated (*r* = 0.54). In contrast, T cells CD8 were negatively related to T cells CD4 memory resting (*r* = −0.67), mast cells resting were negatively related to activated mast cells (*r* = −0.58), and T cells gamma delta negatively correlated to macrophages M2 (*r* = −0.52) (Figure 8(d)). The monocyte and neutrophil counts tend to be higher in AF than SR, while dendritic cells activated and T cells regulatory (Tregs) tend to be lower (*P* < 0.05) (Figure 8(e)).

### 3.8. Correlation Analysis between Hub Genes and AF-Related Infiltrating Immune Cells

**Figure 9:**
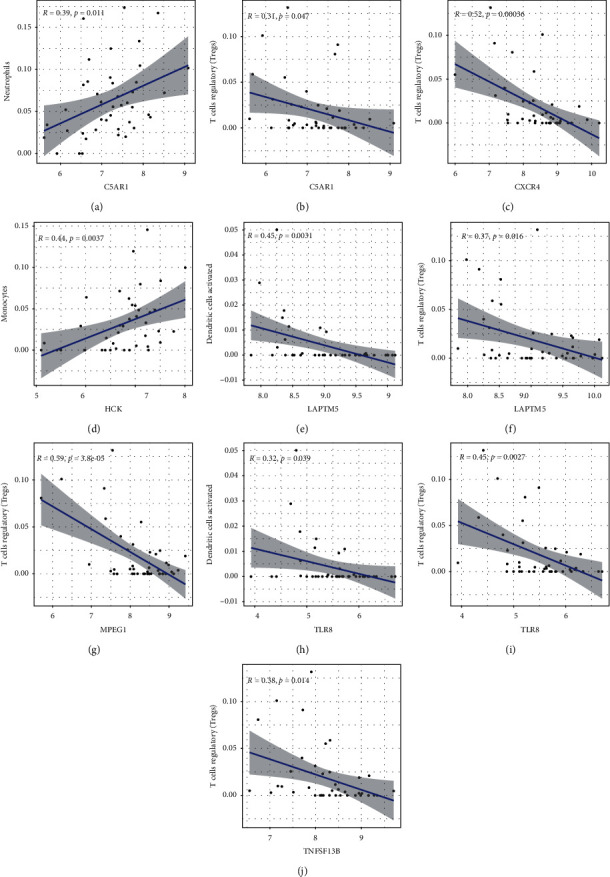
Pearson correlation coefficients were used to calculate the relationship between hub genes and immune cells related to atrial fibrillation (a–j).

A total of 7 hub genes were linearly associated with immune cells associated with AF. C5AR1 was positively associated with neutrophils (*r* = 0.39, *P*=0.011) and associated negatively with T cells regulatory (Tregs) (*r* = −0.31, *P*=0.047). CXCR4 was negatively correlated with T cells regulatory (Tregs) (*r* = −0.52, *P* < 0.001). HCK was positively associated with monocytes (*r* = 0.44, *P* < 0.001). LAPTM5 was negatively associated with dendritic cells activated (*r* = −0.45, *P* < 0.001) and T cells regulatory (Tregs) (*r* = −0.37, *P*=0.016). MPEG1 was negatively associated with T cells regulatory (Tregs) (*r* = −0.59, *P* < 0.001). TLR8 was negatively associated with Dendritic cells activated (*r* = −0.32, *P*=0.039) and T cells regulatory (Tregs) (*r* = −0.45, *P* < 0.001). TNFSF13B was negatively associated with T cells regulatory (Tregs) (*r* = -0.38, *P*=0.014) (Figure 9).

### 3.9. Hub Immune-Related Genes Could be Used to Predict AF Specifically and Sensitively via the ROC Curve Analysis

**Figure 10:**
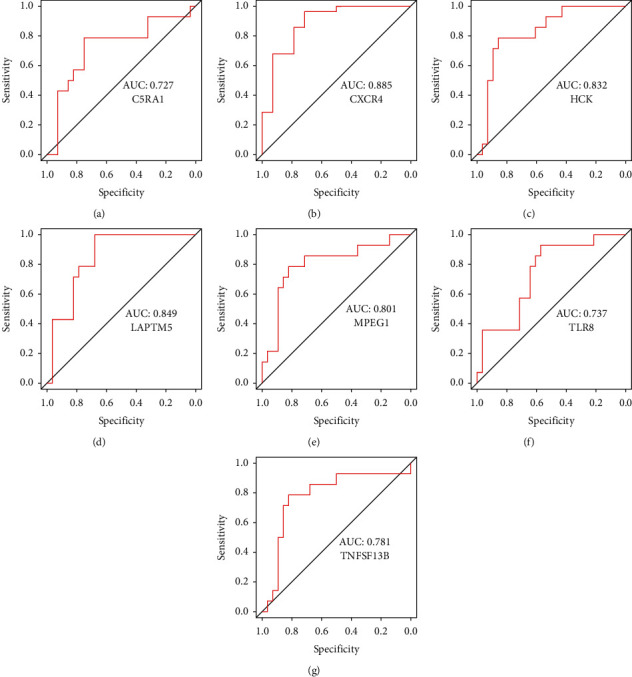
The receiver operator characteristic curves of the hub immune-related genes for atrial fibrillation. (a) C5RA1, (b) CXCR4, (c) HCK, (d) LAPTM5, (e) MPEG1, (f) TLR8, and (g) TNFSF13B.

ROC curve analysis revealed that these seven hub immune-related genes (C5AR1, CXCR4, HCK, LAPTM5, MPEG1, TLR8, and TNFSF13B) were significantly associated with AF diagnosis (0.7 < AUC < 1) (Figure 10).

## 4. Discussion

AF is the most frequent arrhythmia that presents in clinical practice and increases the risk for heart failure, stroke, and death. By further understanding the mechanisms, we may find new strategies to treat AF. We performed a combined analysis of ceRNA networks and immune infiltration associated with AF to explore the molecular mechanism. First, we performed GSEA to analyze the significant difference between AF and SR groups for the integrated data set. Gene sets related to cardiac muscle hypertrophy, collagen binding, innate immune response, chemokine signaling pathway, renin angiotensin system, and T cell receptor signaling pathway were differentially enriched with AF phenotype. The chemokine signaling pathway plays a key role in cardiovascular disease. For example, chemokines and their receptors are important in the recruitment and activation of immune cells and the persistence of the local inflammatory response in atherosclerosis [23]. Chemokines and their receptors have also been shown to be involved in the pathophysiology of cardiac remodeling and heart failure resulting from excessive pressure load [24]. The renin angiotensin system is known to be closely associated with mechanism of AF development. The atrial electrical and structural remodeling are the core part of AF, and the activation of the renin-angiotensin-aldosterone system (RAAS) contributes to atrial remodeling [25]. Conversely, studies have demonstrated that renin-angiotensin system inhibitors can delay heart remodeling in patients with AF [26] and prevent recurrence of AF after ablation [27]. So, GSEA indicated that those gene sets in AF patients were primarily involved in cardiac muscle, immune, and inflammatory responses.

Next, we performed two different methods (WGCNA and LIMMA method) to screen CGs related to AF from the integrated data set. Together with DE circRNAs and DE miRNAs related to AF, the ceRNA network was constructed, suggesting that these mRNAs, miRNAs, and circRNAs in the circRNA-miRNA-mRNA ceRNA network could play an important role in the pathogenesis of AF. GO functional enrichment analysis revealed that these genes in the ceRNA network were significantly involved in the regulation of lymphocyte and mononuclear cell proliferation and immune receptor activity. KEGG pathway analysis results were predominantly enriched in “Chemokine signaling pathway” and “Cytokine–cytokine receptor interaction.” According to GO and KEGG results, ceRNA network about AF in this study was also mainly correlated with inflammation and immunity. Subsequently, we constructed a PPI network based on 55 mRNAs in the ceRNA network and used the MCC algorithm in the CytoHubba plug-in to filter 10 hub genes (CCR5, CXCR4, TLR8, CXCL12, C5AR1, HCK, TNFSF13B, IRF8, MPEG1, and LAPTM5). Among them, the highest scored gene was CCR5. Research has shown that CCR5 is involved in autoimmune and inflammatory diseases such as rheumatoid arthritis [28] by regulating the activation and migration of immune cells. And studies also have indicated that CCR5 may play a role in Ang II-induced hypertension and vascular dysfunction [29], as well as in the development of arthrosclerosis and cardiovascular disease [30]. Additionally, CCR5 inhibition protects against pressure overload-induced cardiac dysfunction through P38 and ERK1/2 signaling pathways [31]. Therefore, we speculated that CCR5 may be implicated in the pathogenesis of AF through immune and inflammatory responses, which needs to be further explored.

Then, CIBERSORT was used to study immune infiltration in AF. According to the study, we found that an increase in monocytes and neutrophils, along with a decrease in dendritic cells and regulatory T cells (Tregs), might be linked to AF pathogenesis. And our bioinformatics analysis showed 7 hub genes (C5AR1, CXCR4, HCK, LAPTM5, MPEG1, TLR8, and TNFSF13B) were associated with those 4 AF-related immune cells, and these genes may be able to predict AF based on the ROC curve analysis (0.7 < AUC < 1). So, we conjectured that these 7 genes may be involved in AF pathogenesis by regulating these 4 immune cells.

Ulteriorly, we performed a combined analysis of circRNA-miRNA-hub gene subnetwork and immune infiltration associated with AF. It has been found that inflammatory infiltration of myocardium, including neutrophils and inflammation markers, may contribute to AF [32]. Neutrophils dominate the inflammatory cells in AF patients who undergo pericardiotomy, atriotomy, or catheter ablation according to some studies [33, 34]. Furthermore, elevated neutrophil-to-lymphocyte ratio (NLR) has been shown to be independently associated with a higher risk of all-cause mortality and combined end point events in patients with AF and to be an independent predictor of long-term prognosis in AF patients [35]. Our study also found neutrophils were upregulated in AF tissues. We found that T cell regulatory (Tregs) proportion in atrial tissues of AF patients was significantly lower, which was similar to the results of Chen et al. [36]. And they validated IL-6-miR-210 inhibits Tregs function by targeting Foxp3 to promote atrial fibrosis. Recently, we downloaded immune genes from the database and constructed the immune cell-related ceRNA subnetwork through bioinformatics analysis, which the results showed that Tregs were also underexpressed in atrial auricular tissue of AF [37]. One more study revealed that Tregs alleviate myocardial fibrosis and cardiac hypertrophy in hypertensive mice caused by angiotensin II [38]. This suggests that neutrophils and Tregs might be important core cell subtypes in driving AF disease progression. Furthermore, our finding showed that C5AR1 was highly expressed in the auricle tissue of AF patients and was positively associated with neutrophils and negatively correlated with Tregs. Studies confirmed that high levels of C5a and its interaction with C5aR1 led to excessive activation of central neutrophil functions [39, 40]. Meanwhile, C5AR1 was involved in cardiovascular disease. C5aR1 activation has been reported within atherosclerotic plaques [41, 42], and C5AR1-deficient mice revealed obviously mitigate cardiac remodeling and inflammation after Ang II infusion [43]. From our constructed circRNA-miRNA-hub gene subnetwork, two downregulated miRNAs (miR-1246 and miR-483-5p) and their upregulated C5AR1 target gene were regulated by four upstream upregulated circRNAs. Study found that miR-1246 in endothelial progenitor cell (EPC)-derived exosomes enhanced in vitro and in vivo angiogenesis in myocardial infarction (MI), and these improvements may be involved in the reduction of myocardial injury and cardiac fibrosis after MI [44]. Therefore, we hypothesized that low expression of miR-1246 in patients with AF may ultimately lead to atrial fibrosis by targeting and enhancing fibrosis-related gene expression. These reports suggested that circ_0005299–miR-1246–C5AR1 regulatory axis could be associated with the immune mechanism of AF.

Previous study has shown that dendritic cells are present in damaged heart tissue and play a significant role in cardiac remodeling after MI [45]. However, we found that activated dendritic cells were downregulated (*P* < 0.05), and resting dendritic cells tended to be upregulated (*P* > 0.05) in the left atrial tissue of patients with AF. We speculated that different timepoints of the AF onset may determine the contrary results.

In AF patients, the proportion of intermediate monocytes was higher compared with the control group [46], especially in those with low-voltage zones [47]. Furthermore, the activation of monocytes, more specifically their enhanced migration ability, is crucial in the pathogenesis of atrial remodeling in AF patients [48, 49]. Our analysis also found that monocytes were highly expressed in atrial tissues of AF patients, and GO enrichment analysis of genes in the ceRNA network was mainly enriched in regulating the proliferation of monocytes, which was positively correlated with HCK gene. Study showed that HCK gene expression was increased in LPS-stimulated human peripheral blood monocyte-derived macrophages [50]. And HCK as critical for regulating alternative activation of monocytes [51]. Our study found that Tregs were also negatively associated with other 5 hub genes (CXCR4, LAPTM5, MPEG1, TLR8, and TNFSF13B) in AF tissues. It has been reported that CXCR4 and LAPTM5 are involved in the negative regulation of Tregs [52, 53]. Moreover, Wang et al. [54] showed that CXCR4 is overexpressed in AF patients, which may lead to the occurrence of AF by modulating atrial fibrosis and structural remodeling. Additionally, the ceRNA subnetwork found that downregulated miR-623 and its upregulated HCK and CXCR4 target genes were coregulated by circ_0006725 and circ_0079284. Ring Finger 216 (RNF216) is the host of has_circRNA_0079284, and study reported that it may be involved in innate immune signaling [55]. Study on intervertebral disc degeneration has shown that miR-623 directly bound CXCL12 to reduce levels of inflammatory factors in LPS-injured nucleus pulposus cells [56]. Those indicated that circRNA_0079284, miR-623, HCK, and CXCR4 may play roles in immune and inflammation. Our result suggested that the interaction between circRNA_0079284-miR-623-HCK/CXCR4 may be associated with the immune mechanism of AF. Other circRNA-miRNA-mRNA regulatory axes composed of these 7 hub genes in the ceRNA subnetwork may also be related to immunity (Figure 6(e)).

However, there are some limitations to this literature. First, only a few samples were used for miRNA and circRNA microarray analysis. Second, not all samples used for miRNA microarray analysis were from left atrial appendage, and tissue samples from different parts of atrial of patients with AF did not achieve homogeneity between samples, which may produce bias to the results. Third, in this study, the genes that predicted AF were derived from tissue samples, and the biomarkers for diagnosing persistent AF need to be tested in blood samples from those patients. Finally, further in vitro and in vivo experiments are needed in the future to validate the results deduced by bioinformatics analysis.

## 5. Conclusion

Through the analysis of immune-related ceRNA networks, our findings provide novel insight into the molecular mechanisms underlying the progression of AF. And we found that the circ_0005299–miR-1246–C5AR1 and circRNA_0079284-miR-623-HCK/CXCR4 regulatory axises may be associated with the immune mechanism of AF.

## Figures and Tables

**Table 1:** Top 10 genes with higher MCC score in protein-protein interaction network.

Gene symbol	MCC score	logFC	*P* Value	Gene title
CCR5	156	0.530	0.0091	C–C chemokine receptor type 5
TLR8	156	0.503	0.0071	Toll-like receptor 8
C5AR1	126	0.508	0.0296	C5a anaphylatoxin chemotactic receptor 1
CXCL12	126	0.584	0.0083	Stromal cell-derived factor 1
CXCR4	120	1.096	1.54E-06	C-X-C chemokine receptor type 4
TNFSF13B	120	0.573	0.0093	Tumor necrosis factor ligand superfamily member 13B
HCK	32	0.693	0.0005	Hematopoietic cell kinase
MPEG1	30	0.783	0.0005	Macrophage gene 1 protein
IRF8	26	0.633	0.0013	Interferon regulatory factor 8
LAPTM5	2	0.730	4.88E-05	Lysosomal-associated transmembrane protein 5

Positive logFC values correspond to upregulated genes. FC, fold change; MCC, maximal clique centrality.

**Table 2:** The basic information of the 9 circRNAs in the ceRNA subnetwork.

CircRNA ID	logFC	*P* Value	Chr	Genomic length	Strand	Gene symbol
hsa_circ_0001615	3.09	0.007	6	411	−	PHIP
hsa_circ_0004390	3.24	0.008	1	754	−	LPAR3
hsa_circ_0004771	3.02	0.006	21	203	−	NRIP1
hsa_circ_0005299	3.73	0.001	3	278	−	SHQ1
hsa_circ_0002103	3.85	0.012	17	691	+	NLK
hsa_circ_0006725	3.14	0.041	5	575	+	RHOBTB3
hsa_circ_0079284	3.55	0.015	7	321	−	RNF216
hsa_circ_0001190	3.31	0.003	21	1568	+	DYRK1A
hsa_circ_0004979	3.06	0.006	18	308	+	ZNF236

Positive logFC values correspond to upregulated circRNAs. Chr, chromosome; FC, fold change.

## Data Availability

The data sets analyzed in this study are available from the corresponding author upon reasonable request.
